# Epigenetic drugs: a novel anti-aging strategy?

**DOI:** 10.3389/fgene.2012.00224

**Published:** 2012-10-31

**Authors:** A. M. Vaiserman, E. G. Pasyukova

**Affiliations:** ^1^D.F. Chebotarev State Institute of Gerontology NAMS of UkraineKiev, Ukraine; ^2^Institute of Molecular Genetics, RASMoscow, Russia

Aging is a natural phenomenon which is peculiar to all living organisms. However, it is believed by some scientists that senescence could be postponed or prevented by certain approaches. Some dietary ingredients and supplements have been suggested to have anti-aging and life-extending effects. Natural and synthetic dietary supplements including anti-oxidants, vitamins, and hormones are among the most popular products on the market, even without solid scientific evidence supporting their efficacy (Olshansky et al., 2002). Recently, a number of synthetic drugs used for various therapeutic applications have also been assumed to have anti-aging potential (Kapoor et al., 2009). In most cases, however, the high expectations for these drugs were not fulfilled. The effects of several substances, e.g., anti-oxidants, have been supported by data obtained in animal models, but when carefully controlled human trials have been conducted, questions about the efficacy and safety of these substances have emerged (Jerome-Morais et al., 2011). Excessive intake of anti-oxidants, vitamins, or hormones is known to destroy delicate control mechanisms of homeostatic balance, and it is not yet clear under which conditions, if any, they may have a long-term beneficial impact on life expectancy in humans (Cochemé and Murphy, 2010). Given this situation, it is necessary to increase the choice of chemical compounds which have the potential to positively affect longevity. In this Opinion, we present arguments that the development of specific drugs which target epigenetic pathways could be a highly promising anti-aging strategy.

Epigenetic factors including DNA methylation, histone modifications, and alteration in microRNA expression play key roles in controlling changes in gene expression and genomic instability throughout the human lifespan. Epigenetic modifications are finely balanced and highly reversible in normal tissues. However, they may be imbalanced and heritable in tumor and other abnormal cells. Epigenetic dysregulation has a causal effect on age-associated disorders including cancer, atherosclerosis, type 2 diabetes, neurodegenerative and psychiatric diseases, and the decline in immune response (Berdasco and Esteller, 2012). There is increasing evidence to indicate that epigenetic mechanisms are intimately involved in synaptic plasticity and are essential for learning and memory. Dysfunction of epigenetic gene expression in the brain may be involved in neurodegenerative and psychiatric diseases (Sananbenesi and Fischer, 2009; Berdasco and Esteller, 2012).

Therefore, there is nothing surprising in the fact that, at present, great expectations in the treatment of diseases are associated with the use of so-called “epigenetic drugs” that can modulate the activity of enzymes capable of causing epigenetic changes. In this context, members of the superfamily of histone deacetylases (HDACs) comprising HDAC 1–11 and sirtuins (SIRT) 1–7 are currently the focus of significant interest (de Oliveira et al., 2012). The potential reversibility of epigenetic aberrations has made them attractive targets for therapeutic intervention. Several drugs which target the epigenetic machinery, such as HDAC modulators, mainly inhibitors, have recently been used in human clinical trials, and some have been recommended for the treatment of age-associated diseases (Gryder et al., 2012; Price et al., 2012; Sato, 2012).

Above all, HDAC inhibitors are considered promising anti-cancer therapeutics (Karagiannis and Maulik, 2012). Recently, HDAC inhibitors have shown anti-tumor activity against certain hematological malignancies; their therapeutic potential in solid tumors remains more uncertain (Gryder et al., 2012). Vorinostat and romidepsin have recently been approved for the treatment of relapsed or refractory T-cell lymphoma in the USA and Japan (Price et al., 2012; Sato, 2012). Numerous studies have identified HDAC inhibitors as candidate drugs for the treatment of neurodegenerative disorders. These agents can ameliorate deficits in synaptic plasticity, cognition, and stress-related behaviors in a wide range of neurologic and psychiatric disorders including Huntington's disease, Parkinson's disease, Alzheimer disease, anxiety and mood disorders, Rubinstein–Taybi syndrome, and Rett syndrome (Abel and Zukin, 2008; Xu et al., 2011). HDAC inhibitors are also a new class of immunomodulatory and anti-inflammatory therapeutics (Akimova et al., 2012; Cantley et al., 2012; Licciardi and Karagiannis, 2012). Recent data identified an essential role for HDAC inhibitors in regulation of the expression of innate immune genes and host defenses against microbial pathogens (Roger et al., 2011). The anti-cancer effects of HDAC inhibitors may also be linked to long-term stimulation of the immune response (Leggatt and Gabrielli, 2012).

In recent years, high hopes have been placed on the therapeutic potential of modulators of NAD-dependent class III histone deacetylases (sirtuins). Sirtuins are becoming increasingly recognized as attractive novel therapeutic targets for metabolic, cardiovascular and neurodegenerative diseases, and cancer (Huber and Superti-Furga, 2011; Carafa et al., 2012). Both activation and inhibition of sirtuins may be useful for preventing and treating age-related diseases, depending on the pathological condition, and the target tissue (Mahajan et al., 2011). A number of sirtuin inhibitors demonstrated anti-proliferative effects in cell assays as well as in mouse tumor models, thus suggesting a possible role in cancer therapy. The selective inhibitor of SIRT2, AGK-2, has been reported to have protective effects against Parkinson's disease, and resveratrol and other sirtuin activators can be useful in the treatment of Alzheimer's disease (Mai, 2010).

HDACs are global transcriptional regulators; they affect gene expression by deacetylation of not only histones but also non-histone proteins, including transcription factors, and are involved in the regulation of signal transduction, cell cycle and cell growth, DNA damage response, apoptosis, and differentiation. Sirtuins have been implicated in determining the balance between apoptosis, cell survival, and cell proliferation, and are also involved in the regulation of metabolism and stress, two key factors that affect the process of aging (Satoh et al., 2011). The therapeutic effects of HDAC inhibitors are based on their ability to affect the transcription of various genes; in particular, anti-tumor effects can be attributed to the transcriptional reactivation of silent tumor suppressor genes and the transcriptional repression of proto-oncogenes (Boumber and Issa, 2011). Overall, as a major mechanism of transcriptional regulation, protein acetylation is a key controller of many physiological processes essential for the maintenance of homeostasis and a healthy lifespan. Consequently, it is believed that the development of specific drugs which target HDAC activity could be a highly promising anti-aging strategy.

Indeed, the efficiency of promising anti-aging dietary compounds, such as resveratrol and curcumin, can by explained, at least partly, by their ability to modulate gene expression via interaction with HDACs. For example, resveratrol has been shown to inhibit HDAC activity in a concentration-dependent manner (Dayangaç-Erden et al., 2009). Albeit the action of resveratrol is complex, the resveratrol-induced SIRT1 activation is believed to be the main reason for its anti-aging effect (Price et al., 2012). Some of the biological activities of curcumin, such as its anti-oxidative, anti-inflammatory, anti-cancer, chemopreventive, and anti-neurodegenerative effects, may be due to its capability to modulate gene expression through its interaction with HDACs, histone acetyltransferases, DNA methyltransferases, and microRNAs (Reuter et al., 2011). For example, in the study by Lee et al. (2011) curcumin-induced apoptosis and cell cycle arrest at the G2/M phase in medulloblastoma cells were accompanied by reduced HDAC4 expression.

Among the chemicals affecting HDAC activity, HDAC inhibitors are obvious candidates for the role of anti-aging agents. Indeed, a decrease in HDAC activity would lead to increased transcription of many genes. With age, the transcription profiles of different genes change in different ways. However, for the majority of genes, primarily metabolic and biosynthetic genes, a decline in transcription is observed in old age (Lee et al., 1999; Seroude et al., 2002). There is hope that HDAC inhibition will promote temporal homeostasis and delay aging due to preservation of the level of transcription, which is characteristic of the young, in aging organisms. In addition, HDAC inhibition may result in up-regulation of inflammatory response and stress response genes—changes that are usually associated with increased longevity (Vermeulen and Loeschcke, 2007; Kourtis and Tavernarakis, 2011).

It still remains an enigma how fine-tuning of the expression of different genes necessary for normal functioning of an organism could be provided in this case. An imbalance in HDAC activity, similar to an imbalance in the supply of anti-oxidants, vitamins, or hormones, can destroy delicate control mechanisms providing homeostasis. However, it is worth mentioning that epigenetic regulation of gene transcription is a highly coordinated process mediated by central regulatory mechanisms, which can take over functions necessary for a proper orchestration of epigenetic interventions. It makes epigenetics an attractive candidate molecular mechanism suited for the control of highly integrated biological process such as aging (Vaiserman, 2011). Alternatively, one can expect that stage-, tissue-, and HDAC-specific inhibitors will be developed. A similar approach is now being implemented with respect to protein kinase inhibitors, aimed at treating various diseases, especially cancer (Lamba and Ghosh, 2012). Certainly, use of epigenetic drugs can have multiple and complex effects on different systems and tissues. For example, one side effect of modulation of epigenetic processes aimed at human life extension could be reduced reproductive activity. However, the reproductive capabilities of modern people can in fact be realized only very partially due to social (but not biological!) reasons. Therefore, such price for long life span does not seem too high.

Notwithstanding all doubts, in recent years, experimental research has emerged on the life-extending potential of synthetic HDAC inhibitors. A substantial increase in both mean and maximum survival by up to 30–50% without diminution of locomotor activity, resistance to stress, or reproductive ability was observed by feeding *Drosophila melanogaster* the HDAC inhibitor, PBA (4-phenylbutyrate), throughout adulthood (Kang et al., 2002). Flies fed PBA showed a global increase in histone acetylation accompanied by a dramatically altered pattern of gene expression of numerous genes, including genes putatively involved in enhancing longevity: superoxide dismutase, elongation factor 1, glutathione S-transferase, cytochrome P450, and three chaperones. All these genes were induced by PBA. Tao et al. (2004) found that the HDAC inhibitor, trichostatin A (TSA), significantly extended the lifespan of flies. Furthermore, TSA promoted *hsp22* gene transcription. In another study conducted by Zhao et al. (2005), the HDAC inhibitors, TSA, and sodium butyrate, were also shown to significantly extend the lifespan and promote *hsp22* and *hsp70* expression in *Drosophila*. In our recent study, flies fed sodium butyrate at concentrations of 10 and 20 mmol/l throughout both pre-adult and adult stages demonstrated significant increases in mean lifespan in both males and females compared with controls; moreover, treatment with 20 and 40 mmol/l sodium butyrate during the adult stage only resulted in a statistically significant increase in male (but not female) lifespan (Vaiserman et al., 2012). The exact molecular mechanisms of these positive effects remain to be elucidated. In particular, it would be good to know whether anti-aging effects of epigenetic drugs in model animals have the same molecular basis as their therapeutic effects in humans.

In conclusion, understanding the molecular mechanisms underlying the protective role of HDAC inhibitors and other modulators of epigenetic processes could bring us closer to the development of novel drug targets for age-associated chronic diseases. In our opinion, this approach may also provide a new way for the development of efficient anti-aging treatments.
